# Nanostructured Ni-Based Alloys as Electroactive Porous Transport Layers for Anion-Exchange Membrane Water Electrolysis

**DOI:** 10.1021/acssuschemeng.5c03298

**Published:** 2025-09-06

**Authors:** Ameya Ranade, Susanta Bera, Vairavel Mathayan, Remco H. M. Timmer, Jordy W. M. Vernimmen, Erwin Zoethout, Hans J. N. van Eck, Mihalis N. Tsampas

**Affiliations:** † Dutch Institute for Fundamental Energy Research (DIFFER), De Zaale 20, 5612 AJ Eindhoven, The Netherlands; ‡ Department of Chemical Engineering and Chemistry, Eindhoven University of Technology, De Rondom 70, 5612 AP Eindhoven, The Netherlands

**Keywords:** plasma irradiation, water splitting, Inconel, Hastelloy, stainless steel, Ni alloys, ionomer-free

## Abstract

Development of efficient, durable, and sustainable materials for anion-exchange membrane water electrolyzers (AEMWEs) is pivotal for producing scalable green hydrogen. This study investigates the use of helium plasma irradiation to fabricate self-supported nanostructures on nickel-based porous transport layers (PTLs), such as nickel, stainless steel, Inconel, and Hastelloy, and evaluates their performance as anodes in AEMWEs. Nanostructuring the PTLs improves their surface properties, such as increased hydrophilicity and higher surface area, leading to an improvement in performance. The Hastelloy PTL as the anode features the highest activity among the tested materials, and an AEM cell using nanostructured Hastelloy PTL as an anode achieves 1 A cm^–2^ at 1.79 V at 50 °C. Furthermore, the cell also shows excellent stability at 1 A cm^–2^ for 500 h with a minimal degradation rate of ∼25 μV h^–1^, indicating the robustness of this material. At elevated temperatures (∼80 °C), the electrolyzer also achieves current densities of ∼2.4 A cm^–2^ at 1.8 V, aligning closely with the technical targets for water electrolyzers. Going forward, the current findings indicate helium plasma treatment as a versatile and eco-friendly approach for fine-tuning the surface morphology of a broad range of materials that can be employed to scale up next-generation AEMWE systems.

## Graphical abstract

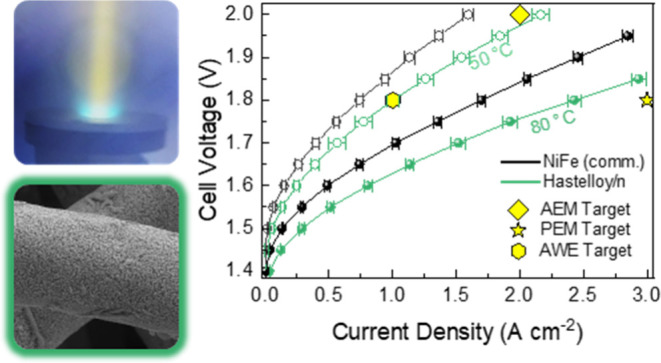

## Introduction

Green hydrogen production via electrochemical water splitting is considered a key technique for seasonal and decentralized energy production, storage, and conversion. Alkaline water electrolysis (AWE) and proton-exchange membrane water electrolysis (PEMWE) are among the leading technologies for low-temperature water splitting.^1^ AWE systems use non-noble electrocatalysts and have higher durability but are limited by slow response to intermittent external load and lower efficiencies.^2^ On the other hand, PEMWE achieves higher performance and is compatible with intermittency but suffers from the use of expensive noble metals and increased corrosion due to the local acidic environment present in the system.^3^ Recently, anion-exchange membrane water electrolysis (AEMWE) has garnered special attention as it can potentially combine the favorable attributes of both AWE and PEMWE. Local alkaline environment enables the use of non-noble electrocatalysts, and a polymer-based anion-exchange membrane can be utilized for achieving high AEMWE performance.^4^

In literature, most AEMWE studies focus on improving membrane properties or developing highly active and stable electrocatalysts.^5–10^ While recent developments in anion-exchange membranes (e.g., Aemion, Sustanion, PiperION) have improved their performance and durability owing to a higher ion exchange capacity and a strong polymer backbone,^11–13^ the development of active and durable electrocatalysts remains a bottleneck in the commercialization of this technology.^14^ Typically, an ink formulation, which involves the emerging electrocatalyst and polymeric binder, is deposited (i) on the membrane to create a catalyst-coated membrane (CCM) or (ii) on the porous transport layer (PTL) to create a catalyst-coated substrate (CCS).^15,16^

A distinct characteristic of the PTLs used in AEMWE is that the commonly employed materials for PTL fabrication (e.g., Ni, Fe, Cr) consist of the same elements that are regarded for their high intrinsic activity and/or synergistic effects in the kinetically sluggish oxygen evolution reaction (OER). In this regard, various grades of stainless steel, Inconel, and Hastelloy (readily available in PTL form) have been recently investigated for their standalone performance.^17–19^ Thus, in addition to (i) mass transport of reactants and products, (ii) heat transport, and (iii) mechanical compression of the cell, the PTLs in AEMWE also serve as the active sites for the OER.

Despite this, most studies primarily report on the overall performance without delineating the inherent performance of the PTLs, leaving a critical gap in understanding their individual roles in AEM water electrolyzers. Recent studies have begun to address this gap by evaluating the performance of standalone nickel-based PTLs as anodes in AEMWE.^20–24^ This approach of using PTLs as electrocatalysts greatly simplifies the manufacturing process, eliminates the necessity of synthesizing separate catalyst powders, and prevent the use of polymeric binders, which are currently a bottleneck in the development of AEM electrolyzers.^25–29^

The inherent activity of PTLs in AEM electrolyzers also opens novel avenues to further optimize their properties to improve the performance. For instance, tailoring the surface properties can have several benefits, such as increased catalyst utilization, reduced interfacial contact resistances, and increased durability.^30,31^ In this regard, various strategies such as fabricating integrated/unified electrodes,^32,33^ developing microporous layers,^34–36^ tuning surface layers via electrochemical methods^37–39^ have been previously investigated.

Recently, helium plasma treatment has emerged as a promising method to fabricate self-supported nanostructures on metallic surfaces while maintaining the material composition.^40,41^ Unlike conventional electrode preparation methods, helium plasma exposure does not generate chemical waste, forms metallic structures at the nanoscale, and is independent of the elemental composition of target materials. While helium plasma irradiation has been primarily studied for the plasma wall interactions in fusion reactors, this method has also been recently applied for fabricating self-supported nanostructures for (photo)­electrochemical water splitting.^42–47^ Here, most studies have focused on improving the (photo)­electrochemical performance using planar two-dimensional (2D) substrates. To the best of our knowledge, this technique has not been applied for nanostructuring three-dimensional (3D) frameworks such as fiber felts, foams, etc.

In this study, we modify the surface morphology of various 3D nickel-based PTLs (nickel, stainless steel, Inconel, and Hastelloy) to fabricate self-supported nanostructures by helium plasma irradiation and evaluate their performance as anodes in AEMWE (schematically presented in Figure 1). The morphology and physiochemical composition of the nanostructured substrates are analyzed by various spectroscopic techniques, while their electrochemical performance and durability are compared in a three-electrode setup and an AEM electrolyzer. This work aims to demonstrate the inherent activity of nickel-based PTLs as anodes and illustrates that surface engineering and functionalization can further improve the performance and efficiency of advanced membrane electrolyzer systems.

**Figure 1:**
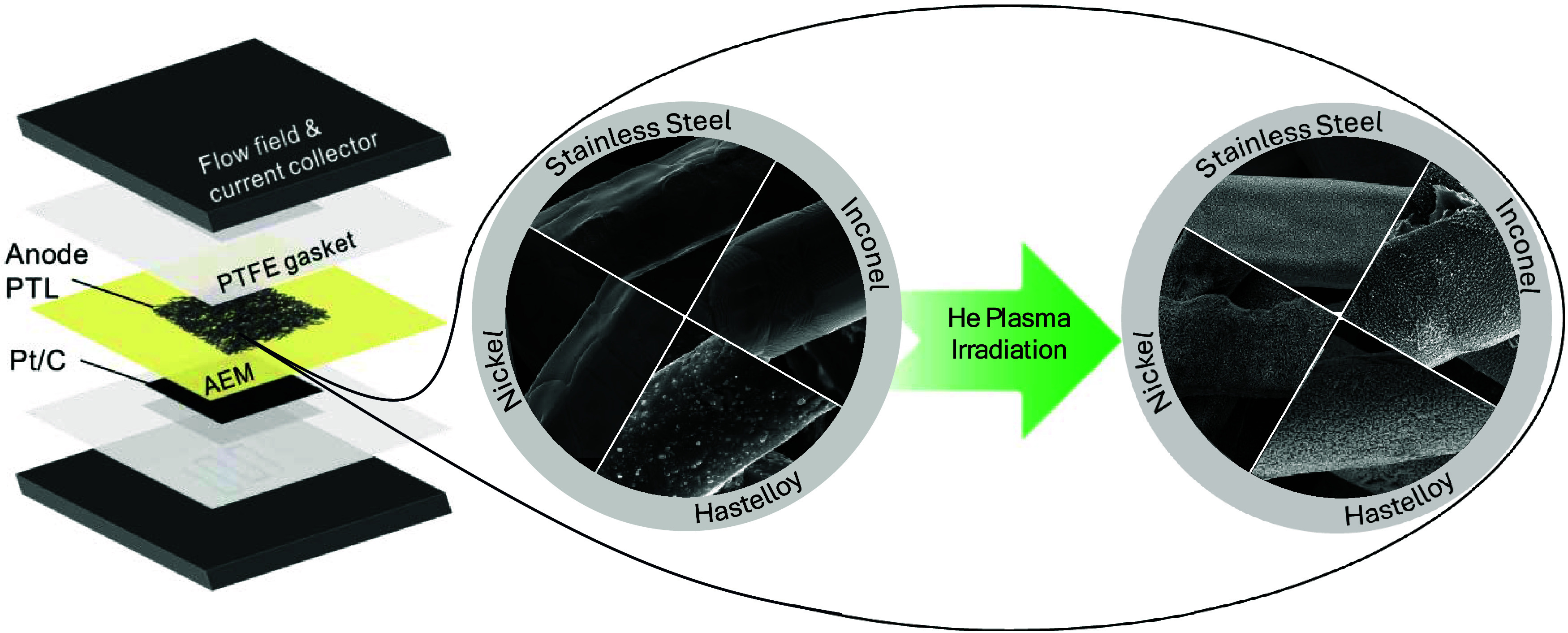
Schematic representation of anion-exchange membrane water electrolyzer (left) and unmodified and plasma-irradiated PTLs (right).

## Experimental Section

### Materials and Chemicals

Nickel, stainless steel, Inconel, and Hastelloy fiber felts (∼78–80% porosity and 0.5 mm thickness, Bekaert) were used as anodes, and 0.5 mg cm^–2^ 60% Pt/C (Fuel Cell Store) was used as the cathode. Planar foils of nickel, stainless steel, Inconel, and Hastelloy (0.01 mm thickness, Goodfellow) were used for determining the contact angle and adsorbate capacitance. KOH (1 M, VWR) was used as the electrolyte in all experiments. PiperION (80 μm, Fuel Cell Store) membranes were first conditioned according to the manufacturer’s instructions before use.

### Nanostructure Fabrication

Nanostructuring on the fiber felts was performed by helium plasma irradiation in a linear plasma generator (Upgraded Pilot-PSI^48^). The helium ions had an incident ion energy of up to 70 eV and a flux of 5–7 × 10^21^ m^–2^ s^–1^. The surface temperature of the fiber felts during the plasma exposure was ∼650 °C, which was recorded with a radiation pyrometer, and the experimental details can be found in a previous study.^45^

### Physiochemical Characterization

Scanning electron microscopy and energy-dispersive X-ray spectroscopy (SEM-EDX; Phenom Pharos Desktop, Thermo Fisher Scientific) were used to determine the morphology and elemental composition of the fiber felts, respectively. A dual-beam scanning electron microscope/focused-ion beam (SEM/FIB; FEI Nova 6001 Nanolab) was used to measure the thickness and uniformity of the nanostructures. X-ray diffraction (XRD; Bruker D8 eco) using Cu Kα with a fixed grazing angle of 1° was used to determine the crystallinity of samples.

Contact angle measurements using a goniometer (Ossila, Sheffield, U.K.) were performed to measure the wettability of the samples. Planar foils (unmodified and nanostructured) were positioned onto the mechanical stage of a contact angle goniometer. A calibrated syringe was used to dispense 7.5 μL of water droplets on the positioned foils. The contact angle measurements were then obtained on the left and right sides of the droplet, which were analyzed by the accompanying Ossila software.

X-ray photoelectron spectroscopy (XPS) was performed to investigate the top composition of the surface of nanostructured PTLs using a monochromatic Al Kα setup from Thermo Fisher Scientific, equipped with a detector at normal incidence. The vacuum environment was maintained at 10^–8^ mbar, and the scans around specific elements were performed with a pass energy of 25 eV. In case of an overlap with Auger peaks of other elements (e.g., Ni LMM Auger peaks overlapping Fe 2p region), a Mg Kα nonmonochromatic source from a SPECS setup equipped with a Phoibos100 detector at normal incidence was used. The pressure was maintained at 10^–9^ mbar, and the scans around specific elements were performed at 50 eV constant pass energy. The binding energy (BE) values of all spectra were referenced to the C 1s line of the adventitious carbon at 284.8 eV.

### Electrochemical Characterization in a Three-Electrode Setup

Electrochemical experiments were performed in a Teflon-based three-electrode setup using an Ivium-n-stat 1A.EIS instrument (Ivium Technologies B.V.). The fiber felts (1 cm × 1 cm; unmodified and nanostructured), graphite rod (redox.me), and Gaskatel Hydroflex RHE (Gaskatel GmbH) were used as the working, counter, and reference electrodes, respectively, and the cell was purged with N_2_. Electrochemical impedance spectroscopy (EIS) was performed at open-circuit potential to evaluate the solution resistance for *iR* correction. The samples were first cycled 10 times from 1.2 to 1.7 V at a scan rate of 50 mV s^–1^ as a pretreatment procedure. EIS measurements at 1.5 V were carried out to calculate the electrochemical surface area (ECSA) of the samples using the double-layer capacitance method. The potential was held for 30 s before each measurement to ensure a steady-state current behavior. The electrochemical performance was evaluated by using cyclic voltammetry (CV) at a scan rate of 20 mV s^–1^. The Tafel slopes and overpotentials were determined by linear sweep voltammetry (LSV; scan rate = 1 mV s^–1^). Each substrate was measured at least two times to test the reproducibility of the results.

### Electrolyzer Assembly and Testing

The electrolyzer setup was operated using an in-house stainless steel cell with serpentine flow fields and a nominal active area of 4 cm^2^. PTFE gaskets were positioned between the MEA components to ensure sealing and prevent any electrical contact. The cell was compressed to a torque of 3.6 N m. The cell temperature was set to 50 °C and controlled by a thermocouple, which was connected to the cell and the thermostat. The anode and cathode sides were fed separately with 1 M KOH electrolyte at a flow rate of 12 mL min^–1^. The electrolyte tanks were degassed with N_2_.

The measurement protocol consisted of a cell conditioning step, wherein the applied current was varied from 50 mA cm^–2^ to 1 A cm^–2^ and then back to 50 mA cm^–2^, with steps of 50 mA. Each step was held for 120 s. Chronoamperometry was also performed over the range of 1.4–2 V, with steps of 50 mV. In addition, chronopotentiometric polarization curves were recorded by stepping the current from 50 mA cm^–2^ to 1 A cm^–2^. EIS measurements were performed between 50 mA cm^–2^ and 1 A cm^–2^, with a frequency range of 50 kHz to 1 Hz and an amplitude of 10% DC. The short-term durability test of the anode PTLs was investigated by chronopotentiometric measurements at 1 A cm^–2^ for 20 h. The long-term durability of the nanostructured Hastelloy PTL was tested at 1 A cm^–2^ for 500 h.

Voltage breakdown analysis (VBA) of the PTLs was performed to determine the contributions of the ohmic, kinetic, catalyst layer, and mass-transfer (residual) resistances.^49^ The ohmic losses were determined from HFR, which was determined via the interpolation of the high-frequency region in the EIS spectra. The kinetic losses were calculated from the Tafel slope and the exchange current density of the electrolyzer. The Tafel slope was calculated using the HFR-free voltage in the current range of 5–50 mA cm^–2^, and the exchange current density was obtained from the x-intercept. In electrolyzer measurements, the “effective” Tafel slope encapsulates the effects from the anode and cathode catalysts. Hence, it provides only insights into the kinetics of the overall system. The catalyst layer resistance (*R*_CL_) was calculated from EIS measurements at 1.4 V using linear fitting and a transmission line model.^50^ The residual resistances, which typically involve mass-transfer resistances and bubble resistances, are clubbed together as mass-transfer resistances.

## Results and Discussion

### Fabrication and Physical Properties of Anode PTLs

The anodic PTLs in this study consisted of nickel, stainless steel, Inconel, and Hastelloy PTLs. Nickel-based alloys generally exhibit good corrosion resistance under alkaline conditions by forming a self-passivating layer. The exact corrosion rates depend on the material composition, presence of gases, temperature, and electrolyte concentration.^51^ For certain grades of stainless steel and Inconel alloys, Cr is added to form a protective oxide film on the surface and hinder further ionic transport. Hastelloy, which contains Mo, is thought to suppress the dissolution of Cr and is beneficial during the repassivation of the film.^52^ Thus, the corrosion rates for alloys in alkaline solutions are typically an order of magnitude lower than nickel due to their specific elemental composition.^53^ Owing to the presence of high-valence transition elements that modulate the surface energy levels along with self-passivating properties, these alloys were selected in the present study to highlight their standalone activity and increased performance by nanostructuring. Exposure to helium plasma results in the nanostructure formation on these surfaces and is generally termed as “fuzz”’. The surface modification induced by helium plasma has been extensively studied in the context of plasma wall interactions for fusion reactors, and the broad consensus has attributed fuzz growth to the formation and coalescence of helium bubbles in the near-surface region.^54,55^ The growth toward a specific morphology depends on the interplay between sputtering and annealing, which in turn depends on the specific element or the material composition of alloys.^47,56,57^ In this study, the plasma conditions were chosen and optimized for 3D frameworks based on our previous work.^45,47^

The morphologies of the various substrates after plasma modification were analyzed by SEM, as shown in Figure 2. Here, nanostructures are observed to have grown directly on the individual fibers. The thickness of the as-formed nanostructures was ∼150–200 nm (Figure S1). Owing to the 3D nature of PTLs, nanostructuring occurs where sufficient plasma exposure happens during the treatment. Under the present conditions, nanostructures form on the top 4–5 fiber layers, which are directly in line during plasma treatment. Deeper layers (not visible by SEM but directly in line with the plasma) might be nanostructured. However, their contribution to the electrochemical performance would be minimal due to higher ohmic losses and limited ionic/electronic transport. Figure S2 exhibits this region using a nanostructured Hastelloy PTL. The elemental composition of the felts, obtained from EDX, is summarized in Table 1. The crystallinity of the modified substrates was investigated by XRD (Figure S3), wherein metallic peaks pertaining to the cubic structure were predominantly visible.

**Figure 2:**
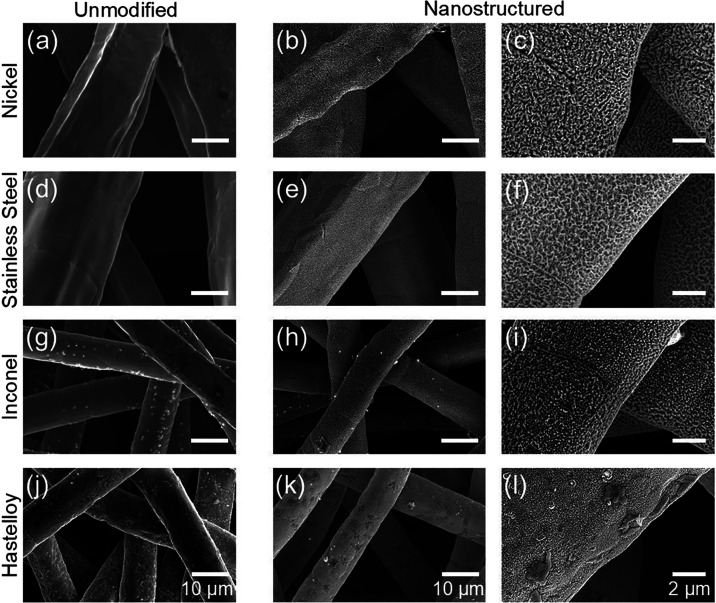
SEM images of the unmodified and nanostructured PTLs. The leftmost column shows the various PTLs in their unmodified state: (a) nickel, (d) stainless steel, (g) Inconel, and (j) Hastelloy. The middle column and right column show the morphology of PTLs after exposure to helium plasma at low and high magnifications.

**Table 1:** Elemental Composition of the PTLs Employed in This Study^a^

	elemental composition (atom %)				
description	Ni	Fe	Cr	Mo	bal.
nickel	100				
stainless steel	8.1	66.2	18.1	0.8	N 2.5, S 1.6, Si 1.5, C 0.7, P 0.5
Inconel	51.4	14.3	24.7		Al 2.9, Cu 2.6, N 1.4 C 1.2, Si 0.8, Ti 0.7
Hastelloy	57.3	2.4	25.7	10.5	N 1.5, C 0.9, Si 0.7, P 0.6

a All PTLs were commercially obtained and had the following features: fiber size, 10–20 μm; thickness, 0.5 mm; porosity, 78–80%. Bal. represents the approximate balance composition of the minority elements.

The surface chemical composition of the nanostructured PTLs was also studied with XPS, and the spectra are shown in Figure S4. It should be noted that the presence of adventitious hydrocarbons and the 3D nature of the nanostructured PTLs affect the surface composition observed via XPS. In the high-resolution Ni 2p spectra, peaks pertaining to metallic nickel species were observed at around 852 and 870 eV in the Ni 2p_3/2_ and Ni 2p_1/2_ region, respectively. In the Fe 2p spectra, weak metallic iron peaks at around 707 eV and oxidic peaks centered at around 711 eV were observed for both stainless steel and Inconel PTLs. The Hastelloy PTL showed no signal in the Fe region before the OER. The Cr 2p spectra of alloy PTLs showed peaks centered at ∼576 eV, indicating chromium­(III) oxides. The Hastelloy PTL also showed the presence of Mo 3d spectra in the metallic and oxidic states. The O 1s high-resolution spectra of all PTLs could be broadly deconvoluted into three regions. The peak at around 530 eV is attributed to the metal–oxygen bond, at around 531 eV to the surface hydroxide, and at 533 eV to various adsorbed species like H_2_O or oxygen-containing hydrocarbons. The aforementioned EDX, XRD, and XPS results and the TEM analysis from previous studies corroborate that the physical composition of the PTLs does not undergo a significant change post plasma exposure^45,47^ and the fabricated nanostructures have a similar elemental composition to the bulk sample. Exposure to ambient conditions results in a thin layer of oxide (native) on the surface, while the bulk remains in the metallic state.

Although the material compositions of the PTLs remained similar after plasma exposure, the physical properties underwent modifications. For instance, the changes in surface tension post-nanostructuring were observed by measuring the contact angle. Planar foils were used for this analysis, as the porous nature of the PTLs made them unsuitable for measuring their surface tension. As seen from Figure S5, the unmodified nickel foil had a high contact angle of 114°. In comparison, the alloys possessed comparatively lower contact angles of 69° for stainless steel, 72° for Inconel, and 81° for Hastelloy. Subsequently, the planar foils were nanostructured using similar parameters from a previous study^45^ to test their surface wettability. All nanostructured foils showcased significantly lower contact angles, with nickel nanostructures having a contact angle of just 5°. The contact angles of nanostructured alloys also reduced considerably to 18° for stainless steel, 10° for Inconel, and 11° for Hastelloy. This decrease in contact angles can be correlated to the increased hydrophilic nature of PTLs.

Studies have shown that the interplay between mass transport of liquid and removal of gas bubbles is better facilitated with an increase in the surface hydrophilicity.^58^ For the present case, as low contact angles of ∼10° indicate superhydrophilicity,^59^ helium plasma exposure appears to result in the fabrication of superhydrophilic surfaces. These surfaces can thus improve the charge and mass transport as well as maintain the bulk material composition while operating the electrolyzers under industrially relevant conditions.

### Performance in a Three-Electrode Configuration

The electrochemical performance of the PTLs was characterized in a Teflon-based three-electrode setup using a 1 M KOH electrolyte. The morphological changes occurring due to plasma irradiation were compared by measuring the electrochemical surface area (ECSA) of the samples. Typically, a specific capacitance of 40 μF cm^–2^ is assumed while calculating the ECSA. However, the specific capacitance (*C*_dl_) is dependent on the material, pH, and applied potential (if any). Hence, assuming one value for different materials can potentially lead to erroneous results. To overcome this issue, we defined a nominal ECSA, which is the ratio of the double-layer capacitance (*C*_dl_) of felts to that of planar foils (calculated by EIS measurements at 1.5 V and fitted using a standard Randles circuit, Figure S6 and Table S1) multiplied by the geometrical area of the foils. The obtained ECSA values via *C*_dl_, given in Figure 3a, are ∼15 cm^2^ for the unmodified samples and ∼20 cm^2^ for nanostructured PTL, representing ∼33% increase by nanostructuring.

**Figure 3:**
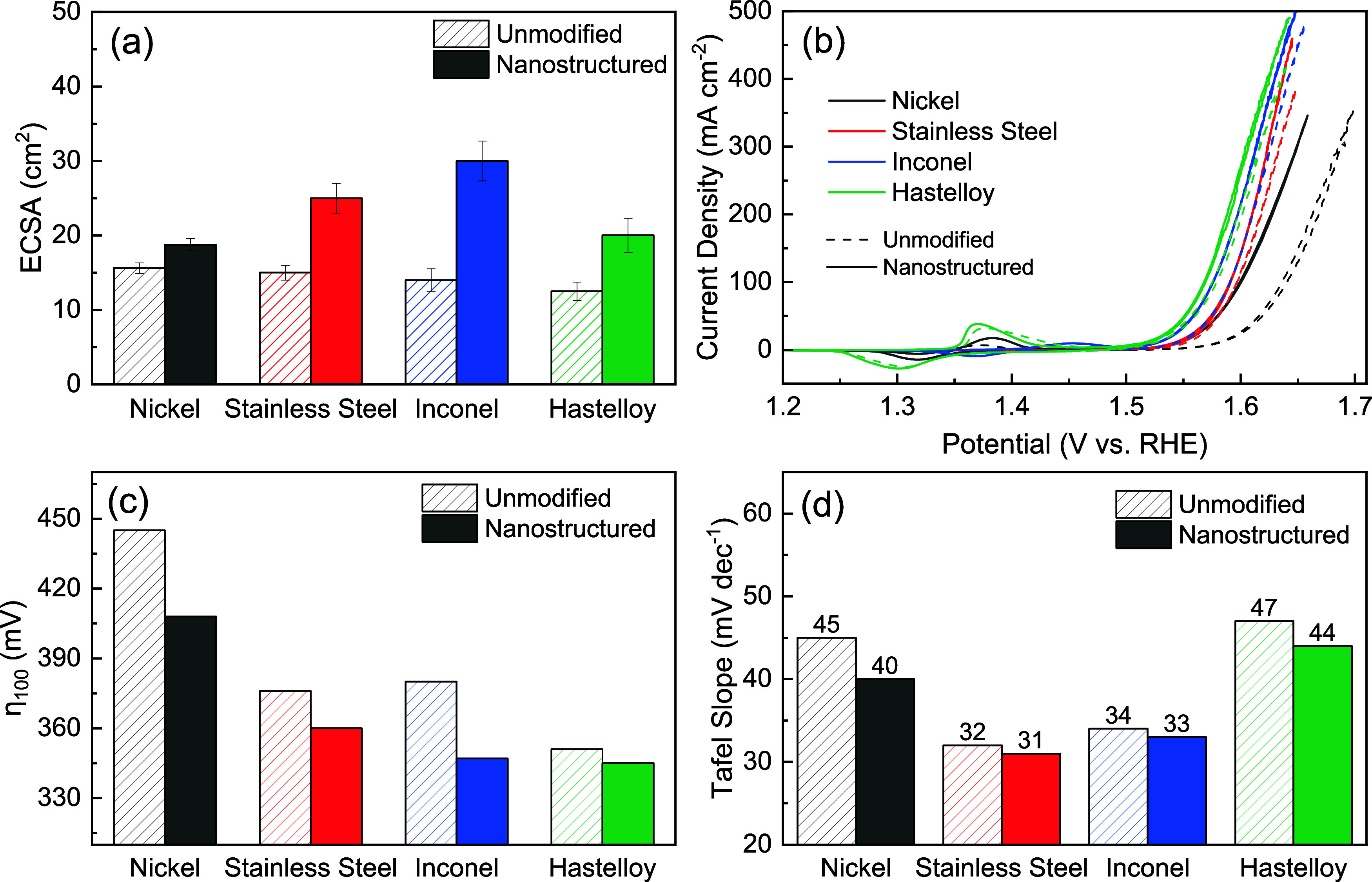
(a) Comparison of the nominal electrochemical surface area (n-ECSA) of the unmodified and nanostructured PTLs using the double-layer capacitance method, (b) CV curves of PTLs, wherein the performance of unmodified PTLs is shown using dashed lines and the performance of nanostructured PTLs is shown using solid lines, (c) comparison of the overpotentials of the unmodified and nanostructured PTLs at 100 mA cm^–2^, and (d) Tafel slopes of unmodified and nanostructured PTLs. All experiments were conducted at room temperature using a 1 M KOH electrolyte.

The electrochemical performances of the unmodified and nanostructured PTLs are shown in Figure 3b. The improvement in the catalytic activity of alloy PTLs toward the OER can be partly attributed to the incorporation of high-valence transition elements in the Ni matrix, which modulate the surface energy levels^60^ and leaching and dissolution of Cr and/or Mo,^61^ increasing the thickness of the Ni (oxy)­hydroxide layer.^62^ The electrochemical activities of unmodified and nanostructured PTLs normalized by ECSA are shown in Figure S7. The overpotentials at 10 mA cm^–2^ showed slight differences (Figure S8) among the various samples. In comparison, the overpotentials at 100 mA cm^–2^, plotted in Figure 3c, show larger variations. The Hastelloy PTLs had the lowest overpotentials of 345 mV for nanostructures and 351 mV for the unmodified PTLs to achieve 100 mA cm^–2^. The overpotentials for Inconel and stainless steel were relatively similar. The nanostructured Inconel PTL required 347 mV, compared to 360 mV for nanostructured stainless steel. For the unmodified samples, an overpotential of 380 mV was needed for Inconel and 376 mV for stainless steel. Nickel PTLs exhibited the largest difference in the overpotential values. The nanostructured PTL achieved 100 mA cm^–2^ at 408 mV, while the unmodified PTL required 445 mV. Since nanostructuring increased the active surface area, a higher amount of iron incorporation would take place, resulting in a higher activity and lower overpotential value. The Tafel slopes for all of the PTLs are shown in Figures 3d and S9. Inconel and stainless steel PTLs possess similar Tafel slopes of ∼30 mV dec^–1^, suggesting similar reaction kinetics over these samples. In contrast, the Tafel slopes of nickel and Hastelloy PTLs were approximately 45 mV dec^–1^. The Tafel slopes of PTLs remained similar, even after nanostructuring. This suggests that the increased activity is mainly from the increased active sites rather than an intrinsic property change or alternate reaction mechanisms.

### AEMWE Performance

The performance of unmodified and nanostructured Ni-based PTLs as anodes (nickel, stainless steel, Inconel, and Hastelloy) was further examined in an AEM electrolyzer. In all cases, 0.5 mg cm^–2^ Pt/C was employed as the cathode, PiperION (80 μm) was employed as AEM, and the cell temperature was maintained at 50 °C. The cell was conditioned prior to data collection, as described in Experimental Section. Figure 4a–d shows the polarization curves of the unmodified and nanostructured PTLs, along with a commercial NiFe catalyst coated on the stainless steel PTL (dioxide materials^11^) for comparison. In all cases, the nanostructured PTLs outperform the unmodified ones, with Hastelloy PTLs showing the highest activity. The overall improvement in performance by nanostructuring could be attributed to (i) decreased interfacial contact resistances, (ii) improved mass transport of reactants and products, and (iii) increased availability of active sites. The contribution of these factors to the overall performance was investigated by performing a voltage breakdown analysis^49^ at an overpotential of 1 A cm^–2^ for the unmodified and nanostructured PTLs (Figures 4e–h and S10). For comparison, the voltage breakdown analysis of the commercial NiFe PTL is provided in Figure S11. The high-frequency resistance (HFR) of the nanostructured PTLs was lower by ∼20% than the unmodified ones, resulting in a lower ohmic drop. The kinetic losses accounted for the highest contribution of overpotentials, representing an ∼66% share of the total losses. Considering individual PTLs, the lower intrinsic activity of nickel and stainless steel led to similar kinetic losses. In contrast, higher intrinsic activity by 3d transition elements in Inconel and Hastelloy resulted in lower kinetic losses. Even though Hastelloy shows slightly higher Tafel slopes, a higher current intercept indicates a higher intrinsic activity.^24,62^ Interestingly, the kinetic losses in the nanostructured PTLs showed only ∼10% improvement, suggesting only trivial changes in the reaction kinetics by nanostructuring. It should be noted that the kinetic overpotentials assume negligible cathodic overpotential (Pt/C as the cathode). However, the reader should be aware that this approximation might not be applicable to all material combinations. The losses due to catalyst layer resistances (*R*_CL_) highlighted the role of morphology and catalyst conductivity. Here, the ability of helium plasma to tune the morphology of the substrate and form metallic nanostructures resulted in ∼20% lower losses for the nanostructured PTLs. Finally, the superhydrophilicity of the nanostructures (Figure S5) assists in facilitating uniform distribution of reactants, reduction of local dry zones, and efficient bubble release,^63^ resulting in lower mass-transfer (MT) resistances for nanostructured PTLs.

**Figure 4:**
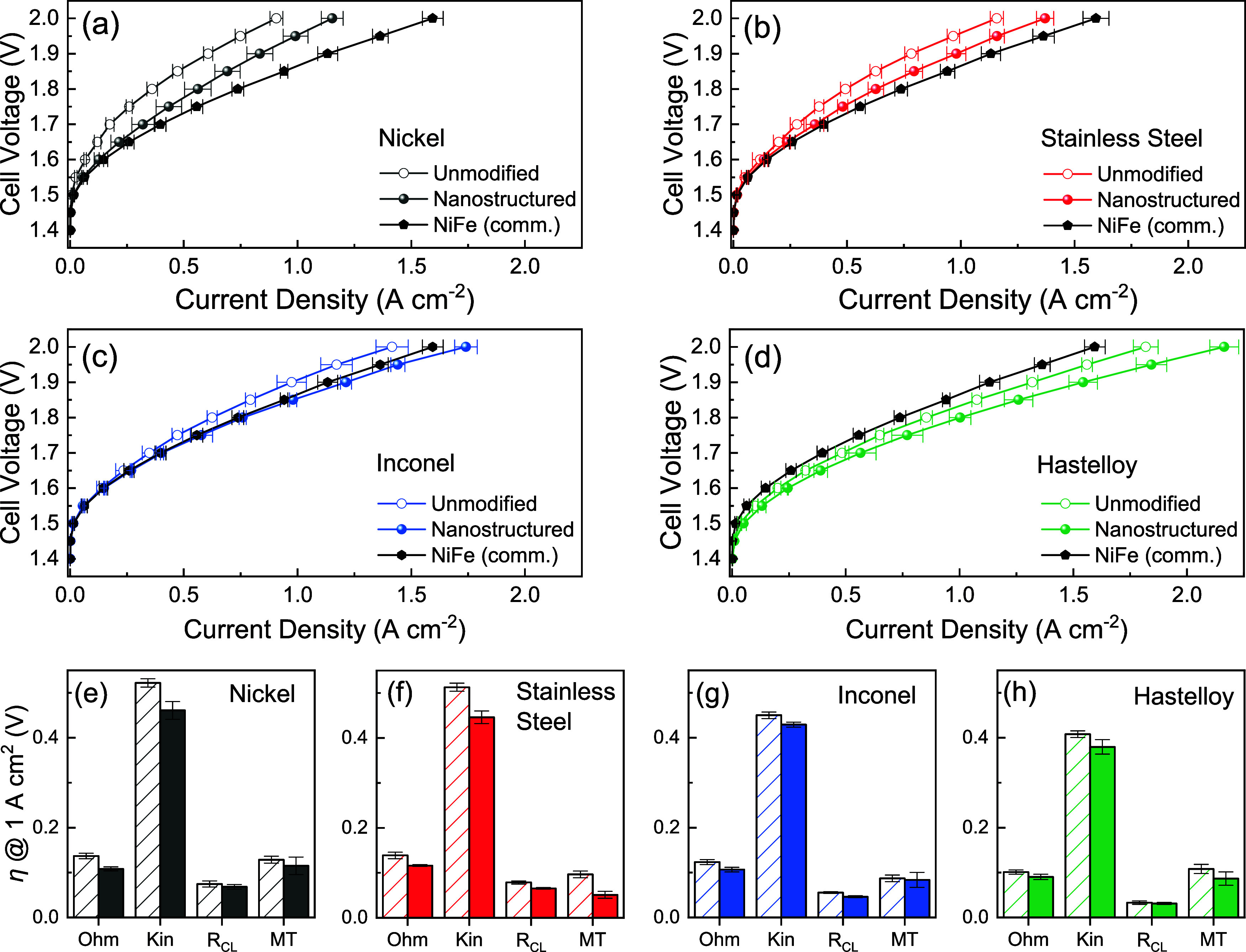
(a–d) Polarization curves of the unmodified and nanostructured PTLs and (e–h) voltage breakdown analysis^49^ of unmodified (line pattern) and nanostructured (solid colors) PTLs at 1 A cm^–2^. Reported data are the average of at least two measurements, and the error bars represent one standard deviation. In all experiments, 0.5 mg cm^–2^ Pt/C was used as the cathode, PiperION (80 μm) was used as AEM, 1 M KOH was separately fed to the anode and cathode compartments, and the cell temperature was maintained at 50 °C.

In summary, the voltage breakdown analysis indicated that the increased activity from nanostructured PTLs can be primarily attributed to the lowering of interfacial contact resistances and catalyst layer resistances. However, the high share of kinetic losses (>66%) further underscores the necessity to develop active and durable electrocatalysts for alkaline environments.

Chronopotentiometric measurements were also performed at 1 A cm^–2^ for 20 h to test the short-term durability of the nanostructured PTLs, as shown in Figure 5a. The nanostructured Hastelloy PTL showed the best durability over 20 h, stabilizing to a degradation rate of ∼0.8 mV h^–1^. This was followed by the NiFe (comm.) PTL and the nanostructured Inconel PTL, which exhibited degradation rates of 2.3 and 2 mV h^–1^, respectively, in the first 20 h. Although Inconel and NiFe (comm.) PTLs showed a similar performance in the beginning, a faster degradation of ∼70 mV was observed for the case of Inconel PTL within the first 5 h. The nanostructured stainless steel PTL also exhibited a low degradation rate of 0.6 mV h^–1^, while the nanostructured nickel PTL showed the highest degradation rate of ∼3 mV h^–1^ and was accompanied by an initial rapid degradation of ∼80 mV in the first 5 h.

**Figure 5:**
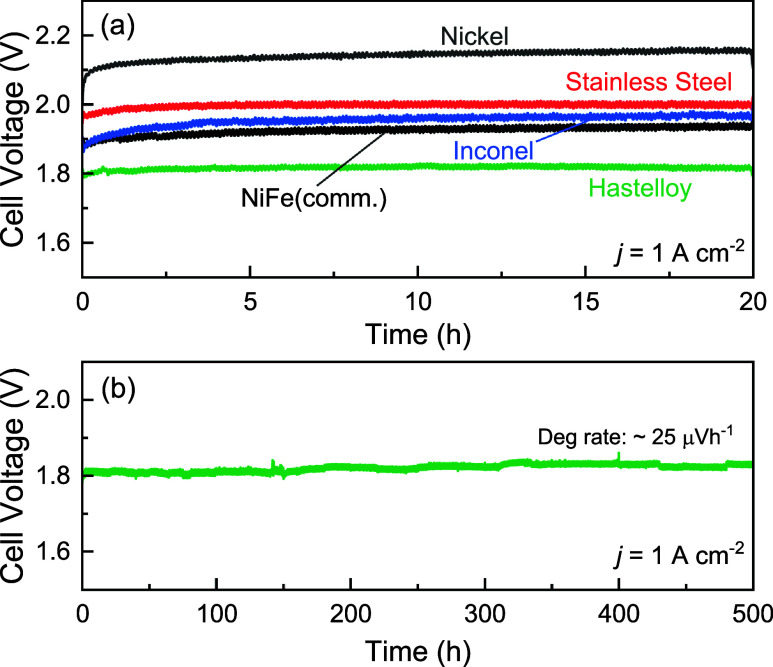
(a) Durability testing of nanostructured PTLs investigated in this work at 1 A cm^–2^ for 20 h at 50 °C. (b) Cell voltage of the nanostructured Hastelloy PTL during the long-term stability test at 1 A cm^–2^ for 500 h. 1 M KOH is used as the electrolyte, and the temperature of the cell was maintained at 50 °C.

Although 20 h is a short duration to test the stability of an electrolyzer, sufficient insights can be obtained to screen the initial performance characteristics of the different anodic PTLs. As Hastelloy had the most promising activity and stability among the tested PTLs, a nanostructured Hastelloy PTL was further tested for 500 h at 1 A cm^–2^, as shown in Figure 5b. The long-term stability indicated that after an initial break-in period (∼50 h), the cell voltage stabilized at around 1.82 V, and the average degradation rate was ∼25 μV h^–1^. Small perturbations in the cell voltage were observed at certain instances, which could be attributed to the temperature fluctuations from the thermostat or a disruption in the flow of electrolyte. The postdurability performance of the Hastelloy nano-PTL was also evaluated and is shown in Figure S12. Only a small increase (∼20 mV) was observed at 1 A cm^–2^, indicating the robustness of the nanostructured PTL. The faradaic efficiency was also tested by comparing the experimental oxygen production rate to the theoretical values at current densities from 0.25 to 1.5 A cm^–2^ and is shown in Figure S13. Here, efficiencies close to 100% indicate that all of the current is utilized for oxygen production and not for any side reactions. It should be noted that during long-term operation, the electrode can undergo various changes such as higher oxidation states, metal dissolution, and surface restructuring.^64^ At present, it is difficult to ascertain the impact of surface reconstruction, the influence of Cr/Mo on self-passivating properties, and material aging of the electrode. Future research could combine techniques such as ICP-MS (to track the composition of dissolved ions) and XAS/XPS (to observe the surface oxidation changes) to investigate electrode corrosion at industrially relevant current densities. These can provide valuable insights for implementing Ni-based alloys in AEM water electrolyzers.

Spectroscopic analysis of the nanostructured PTLs was also carried out after the durability test of 20 h in the AEM electrolyzer. The extent of restructuring and/or morphological changes to the nanostructured PTLs was analyzed via SEM. Figure S14 shows the top-view and cross-sectional views of the nanostructures post-OER measurements in the electrolyzer. The morphology and the thickness of the nanostructures appeared to remain similar, suggesting minimal leaching/detachment of the active catalyst layer. Sheetlike features were observed on the surface, which could be linked to the growth of (oxy)­hydroxide layers formed during oxidative reactions.

The surface composition of the nanostructured PTLs post-OER was measured by using XPS (Figure S15). A broad peak at ∼856 eV in Ni 2p high-resolution spectra indicates that the surface nickel species were predominantly in the (oxy)­hydroxide state for all PTLs. The oxidized surface of PTLs was also corroborated from the O 1s spectra, which mainly showed a metal oxide/hydroxide peak at ∼531 eV. The presence of iron from the electrolyte or as a constituent alloy element was also observed on the surface of all PTLs post-OER measurements. Fe impurities are thought to initially incorporate at the edges/defects and further into the bulk upon potential cycling.^65,66^ The Fe 2p spectra of all PTLs exhibited broad peaks at around 712 and 725 eV, suggesting Fe in 2+/3+ oxidation states. Iron incorporation was observed on nickel PTL, with approximately <1% Fe. For Fe-rich stainless steel, the Ni-to-Fe ratio after the OER was roughly 1:1, suggesting that dissolution and leaching of iron, along with subsequent enrichment of nickel, takes place on the surface.^67^ In contrast, the XPS ratio of Ni:Fe for the Ni-rich alloys (Inconel and Hastelloy) was ∼10:1. The alloying elements in Ni-rich samples (Cr and Mo) play an important role in creating a thicker Ni-rich layer by surface reconstruction, which results in a higher Ni/Fe ratio and a higher activity.^68^ Furthermore, Cr is also known to increase the general corrosion resistance by forming a protective oxide film, which acts as a kinetic barrier to further oxidation and corrosion, while the role of Mo is debated.^61,69^ The dissolution of metals was further confirmed from the Cr 2p spectra of the alloy PTLs, wherein a small peak around 576 eV indicated the Cr­(III) oxidation state. However, the total contribution remained at ≤1%. The Hastelloy PTL also showed peaks in the Mo 3d region at around 231 and 233 eV, suggesting Mo­(IV) and Mo­(VI) oxidation states, respectively. However, even in this case, the overall contribution was only ∼3 atom %. Even though the surface composition changes, the bulk elemental composition does not undergo significant deviations, as evidenced by EDX measurements (Table S2).

### Electrolyzer Performance at Elevated Temperatures

The incumbent water splitting technologies, such as AWE and PEMWE, typically operate at ∼80 °C owing to improved reaction kinetics and electrolyte conductivity. However, AEMWE suffers from the long-term stability of anion-exchange membranes at >60 °C.^70^ Nonetheless, to obtain a direct comparison with established systems (i.e., AWE and PEMWE), as well as AEM studies conducted at higher temperatures, only the AEM cell performance (and no long-term stability tests) using nanostructured Hastelloy PTL was investigated at 80 °C. To avoid membrane degradation, the temperature was first increased to 75 °C and then slowly raised to 80 °C. Active monitoring was done to minimize any overshoot.

The polarization curves in Figure 6 show the temperature dependence of nanostructured Hastelloy PTL at 50 and 80 °C. As expected, the increase in performance became apparent at higher temperatures. The electrolyzer with nanostructured Hastelloy PTL as the anode and 0.5 mg cm^–2^ Pt/C as the cathode achieved a current density of 1 A cm^–2^ at just 1.63 V at 80 °C, compared to 1.79 V at 50 °C. In contrast, the NiFe (comm.) sample could only achieve 1 A cm^–2^ at 1.7 V at 80 °C and 1.89 V at 50 °C. Moreover, the MEA using the nanostructured Hastelloy PTL achieved ∼2.4 A cm^–2^ at 1.8 V at 80 °C (vs 1.7 A cm^–2^ for NiFe (comm.)), thus surpassing the AEMWE target (>2 A cm^–2^ at <2 V), exceeding the 2026 target for AWE (1 A cm^–2^ at 1.8 V), and approaching the 2026 PEMWE target (3 A cm^–2^ at 1.8 V).^71–73^ The performance of unmodified and nanostructured Hastelloy PTL was also compared with that of the literature (Figure S16). The efficacy of the cell at elevated temperatures demonstrates the necessity for developing durable anion-exchange membranes and investigating strategies for mitigating the degradation of auxiliary cell components.

**Figure 6:**
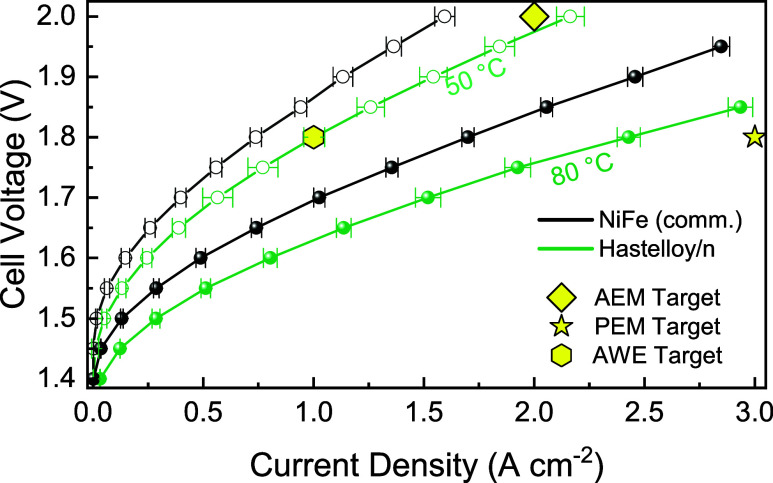
Polarization curves of the nanostructured Hastelloy PTL and NiFe (comm.) PTL at 50 °C (open circles) and 80 °C (closed circles) using 1 M KOH electrolyte. The 2026 technical targets for alkaline water electrolysis (AWE) are given as a yellow hexagon and a yellow star for PEM electrolysis, and the technical target for AEM electrolysis is given as a yellow diamond.

Going forward, as various Ni-based PTLs are extensively used commercially, they present a promising approach to implement them in PTL form for achieving high AEMWE activity and stability. Fine-tuning the structure and material composition can be based on three main parameters: (i) presence of transition elements like Cr/Mo that form a passivating layer under corrosive environments and hinder further ionic transport can improve stability, (ii) transition elements can also leach and dissolute, leading to a higher surface area and consequently a higher activity,^23,67^ and (iii) presence of high-valence transition elements can also modulate the surface energy levels for OER.^60^

## Conclusions

In summary, commercial Ni-based porous transport layers were nanostructured via helium plasma irradiation and were investigated as anodes in an anion-exchange membrane water electrolyzer. Overall, the alloys with the incorporated high-valence 3d transition elements showed improved activity and durability, with Hastelloy showcasing the most promising results. The self-supported nanostructures fabricated by helium plasma resulted in (i) decreased interfacial contact resistance between the membrane and the electrode in an MEA, (ii) improved charge and mass transfer due to their superhydrophilicity, and (iii) additional active sites for reactions, leading to an overall improvement in performance. An AEM electrolyzer employing nanostructured Hastelloy PTL as the anode achieved 1 A cm^–2^ at 1.79 V at 50 °C and had excellent stability for 500 h, with the average degradation rate being ∼25 μV h^–1^. Furthermore, at elevated temperatures (∼80 °C), the electrolyzer also achieved current densities exceeding 2.4 A cm^–2^ at 1.8 V, aligning closely with the technical targets for water electrolyzers.

This study provides critical insights into the role of PTL surface modifications that have a considerable impact on the performance of water electrolyzers. Particularly, the unified nature of anodes in AEMWE to act as PTLs and electrocatalysts provides unique opportunities to fabricate microporous layers and tailor the surface composition to boost the performance and lifetime of next-generation water electrolyzers. The ability of helium plasma to modify the surfaces of even corrosion-resistant alloys like Inconel and Hastelloy provides a promising and sustainable alternative for fabricating enhanced PTLs that can be incorporated in advanced membrane electrolyzer technologies.

## Supplementary Material



## Data Availability

The data that support the findings of this study is available via a Zenodo repository.

## Notes

Supporting data, additional figures including electron microscopy, focused-ion beam, contact angle measurements, X-ray photoelectron spectroscopy, diffraction patterns, Tafel plots, and faradaic efficiency measurements (PDF)
