# Supplementary material for: Nanostructured Ni-Based Alloys as Electroactive Porous Transport Layers for Anion-Exchange Membrane Water Electrolysis
Source: ACS Sustain Chem Eng. 2025 Sep 6;13(37):15291–301. doi: 10.1021/acssuschemeng.5c03298 (PMC12458983; doi:10.1021/acssuschemeng.5c03298)
Supplement: Supplementary file 1 [file sc5c03298_si_001.pdf]

Supporting Information

Nanostructured Ni-based Alloys as Electroactive Porous Transport Layers for Anion Exchange Membrane Water Electrolysis

Ameya Ranade[a,b]*, Susanta Bera[a], Vairavel Mathayan[a], Remco H. M. Timmer[a], Jordy W.M. Vernimmen[a], Erwin Zoethout[a], Hans J. N. van Eck[a], Mihalis N. Tsampas[a,]*

[a] Dutch Institute for Fundamental Energy Research (DIFFER), De Zaale 20, 5612 AJ, Eindhoven, the Netherlands
*Corresponding author: m.tsampas@differ.nl

[b] Department of Chemical Engineering and Chemistry, Eindhoven University of Technology, De Rondom 70, 5612 AP Eindhoven, the Netherlands
*Corresponding author: a.ranade@tue.nl

Supporting Information Content

- 16 Pages
- 16 Figures
- 2 Tables

Table of Contents

1. **Tables**
   a. **Table S1.** Double-layer capacitances of Ni-based foils at 1.5 V
   b. **Table S2.** Elemental composition of PTLs after OER

2. **Figures**
   a. **Figure S1.** Cross-sectional view of the plasma irradiated PTLs
   b. **Figure S2.** SEM image of nanostructured Hastelloy PTL showcasing the approximate region of nanostructuring
   c. **Figure S3.** XRD of plasma irradiated PTLs
   d. **Figure S4.** XPS measurements of nanostructured PTLs before OER.
   e. **Figure S5.** Contact angle measurements of planar and nanostructured foils
   f. **Figure S6.** EIS at 1.5 V of Ni-based foils for double layer capacitance estimation
   g. **Figure S7.** CV curves of PTLs normalized by ECSA
   h. **Figure S8.** Overpotentials (η) of the unmodified and nanostructured PTLs at 10 mA cm^{-2}
   i. **Figure S9.** Comparison of the Tafel slopes of the unmodified and nanostructures PTLs
   j. **Figure S10.** Voltage breakdown analysis of unmodified and nanostructured PTLs
   k. **Figure S11.** Voltage breakdown analysis of commercial NiFe oxide coated on Stainless Steel PTL
   l. **Figure S12.** Polarization curve of nanostructured Hastelloy PTL before and after 500 h stability test
   m. **Figure S13.** Faradaic efficiency measurement for OER with nanostructured Hastelloy PTL anode
   n. **Figure S14.** SEM images of nanostructured PTLs after OER.
   o. **Figure S15.** XPS measurements of nanostructured PTLs after OER
   p. **Figure S16.** Comparison of the performance of standalone PTLs at 1.8 V using 1 M KOH electrolyte and 80 ˚C cell temperature

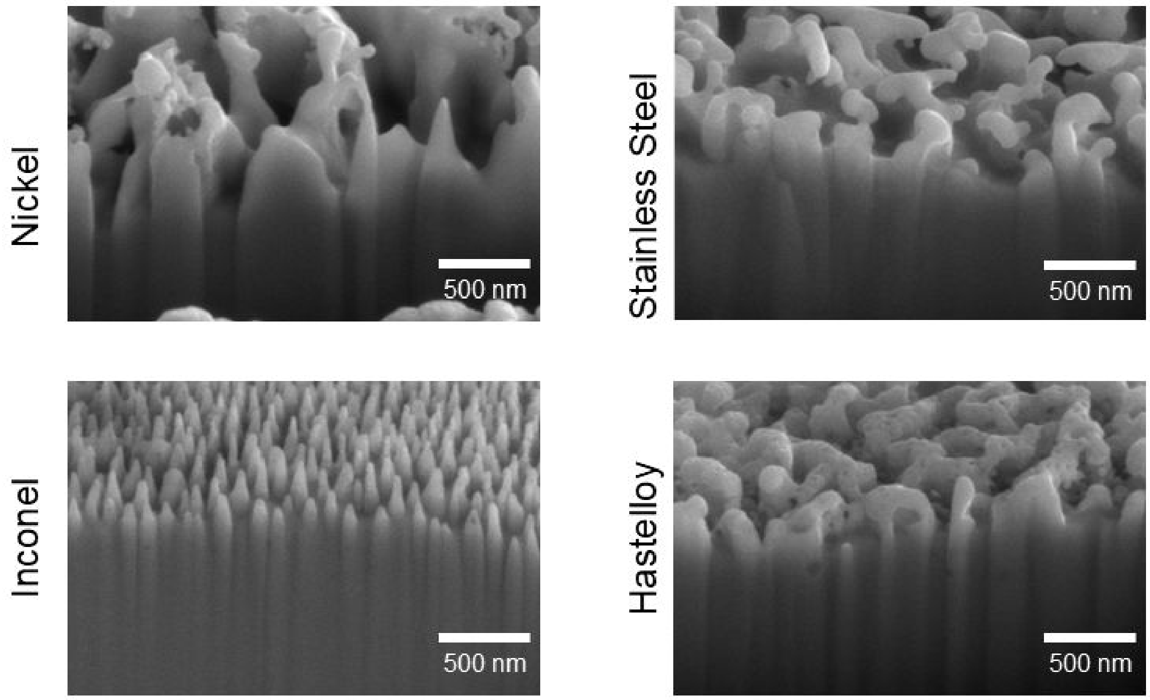

Figure S1: Cross-sectional view of the plasma irradiated PTLs for determining their thickness

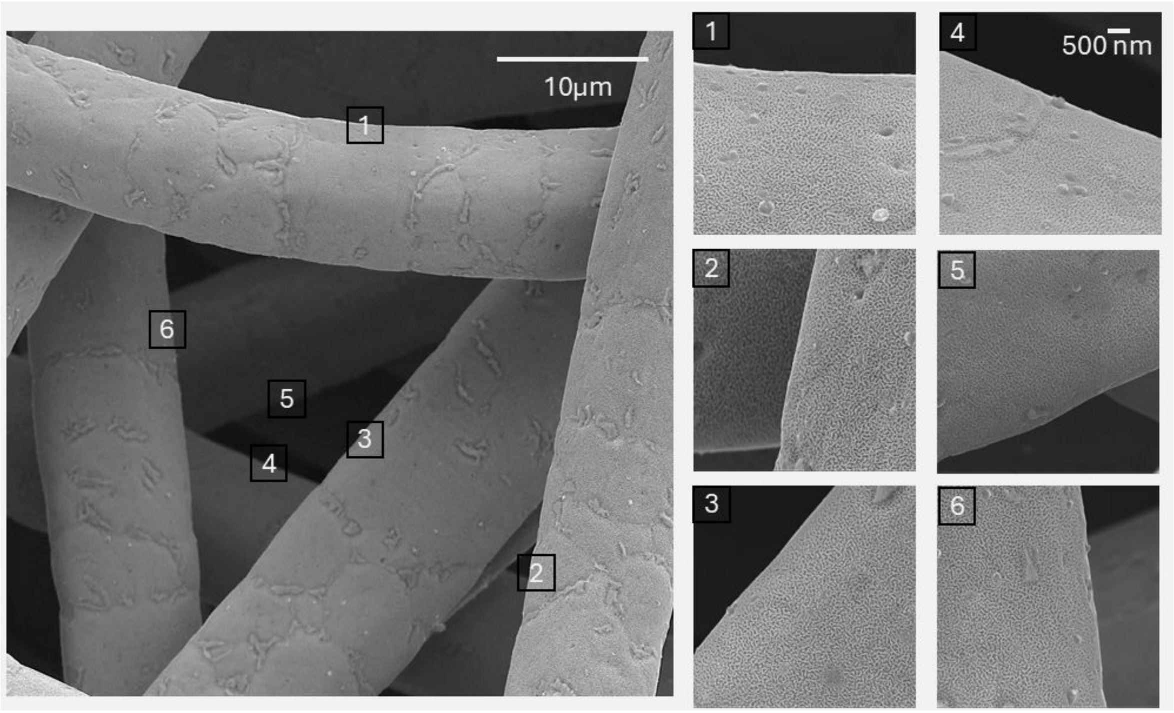

Figure S2: SEM image of nanostructured Hastelloy PTL showcasing the approximate region of nanostructuring. Nanostructuring can happen in deeper layers (not visible by SEM) that are directly in line with plasma. However, their contribution to the electrochemical performance is minimal due to higher ohmic losses and limited ionic/electronic transport.

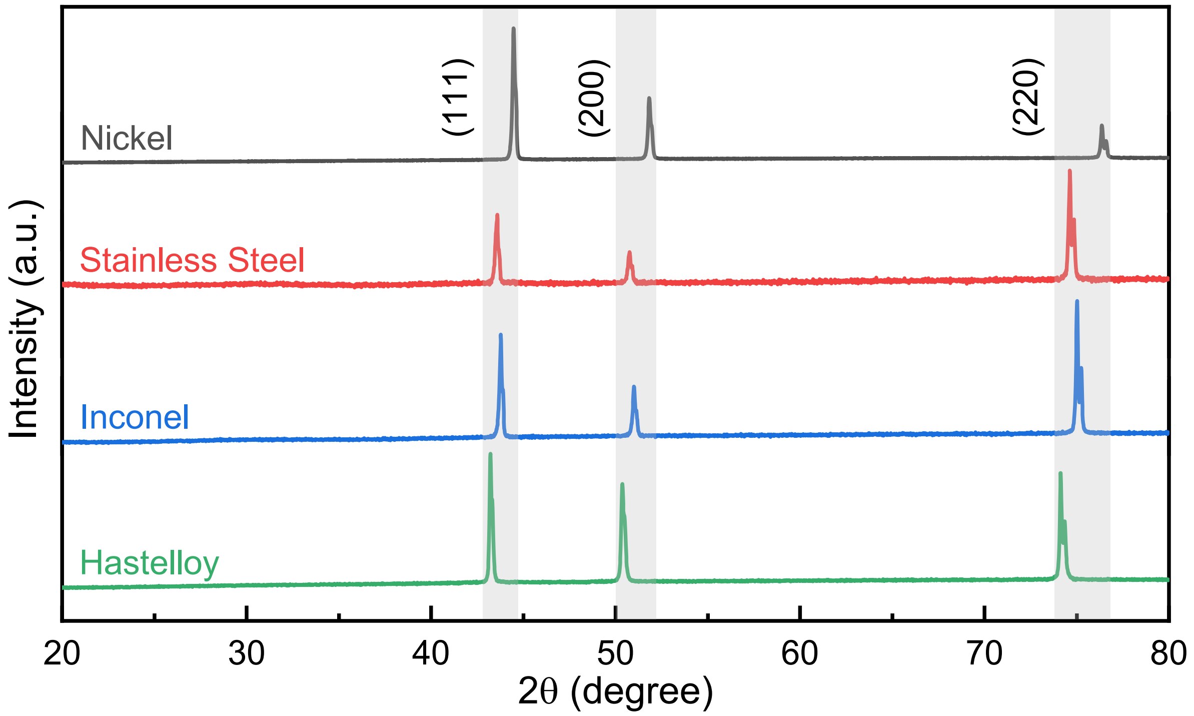

Figure S3: XRD of plasma irradiated PTLs

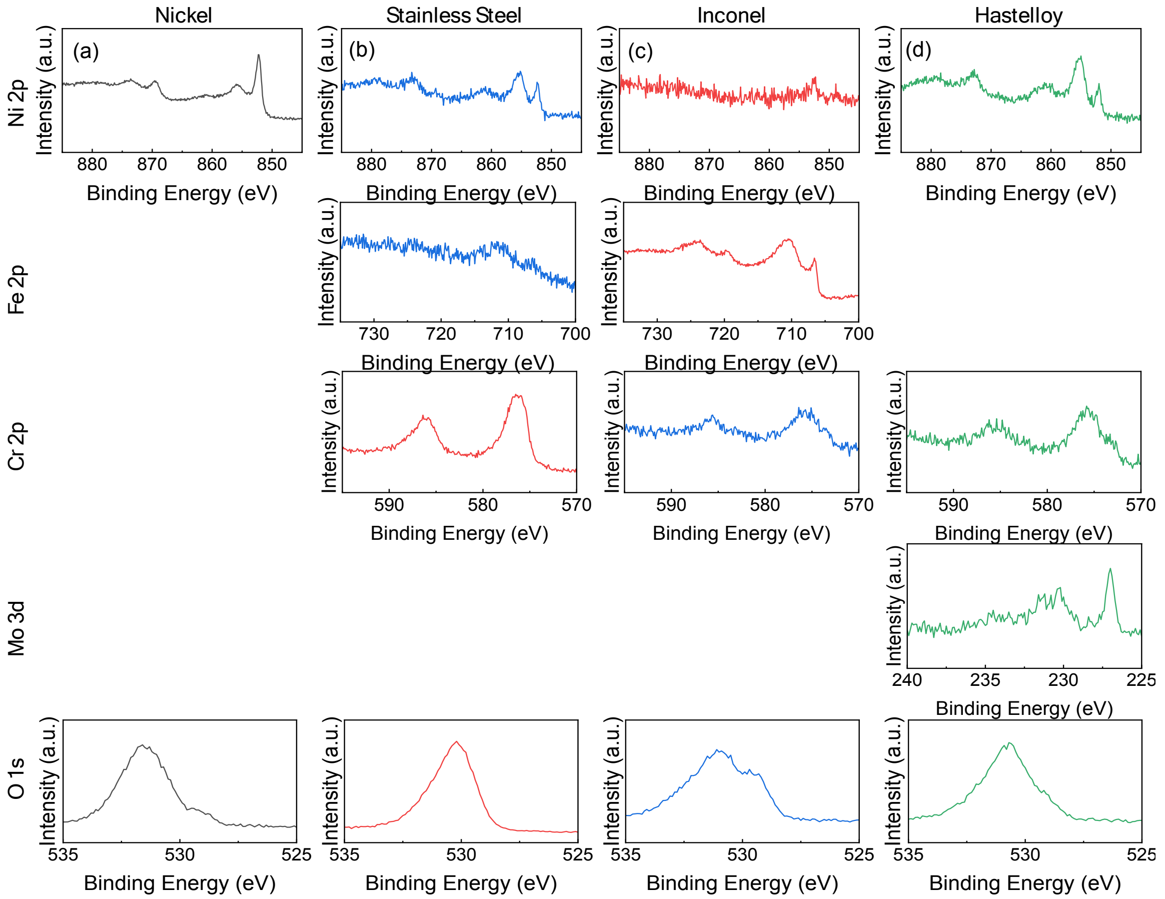

Figure S4: XPS measurements of nanostructured PTLs before OER. The PTL description is given in columns a-d, and the respective spectra are denoted on the left side.

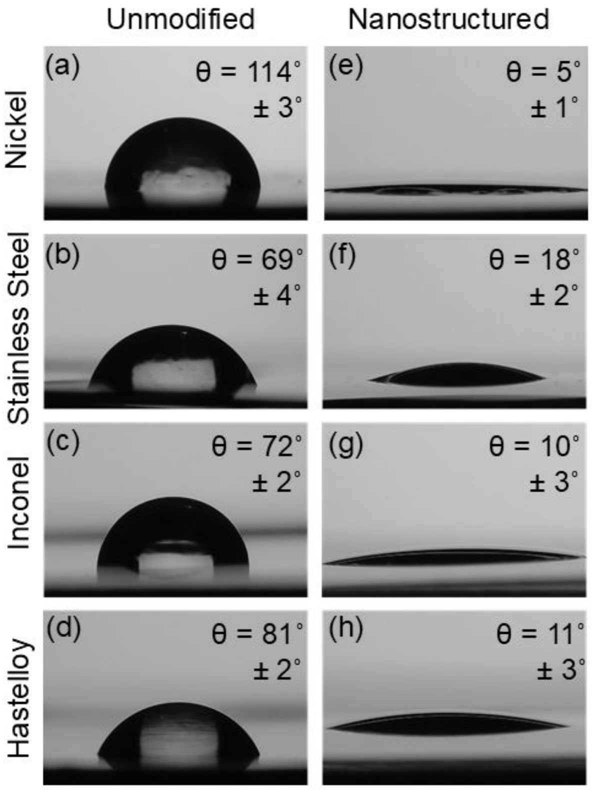

Figure S5: Contact angle measurements of planar foils (a-d), and the plasma irradiated foils (e-h)

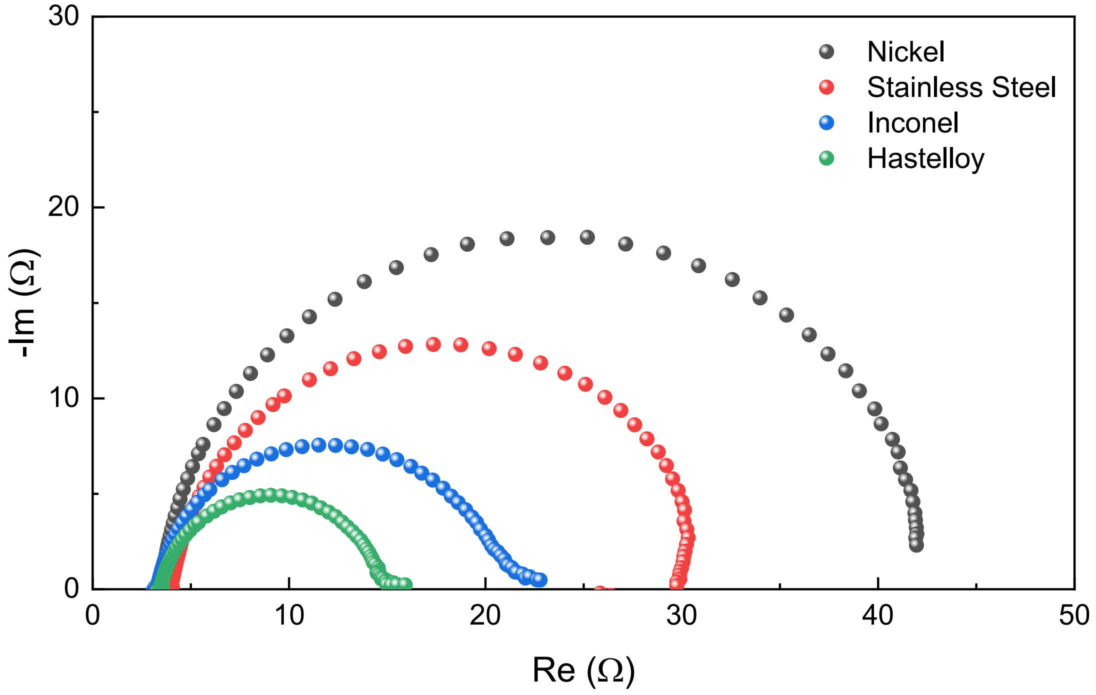

Figure S6: EIS at 1.5 V of Ni-based foils for double layer capacitance estimation

Table S1: Double-layer capacitances of Ni-based foils at 1.5 V

Description	Double-layer capacitance (mF cm^{-2})
Nickel	0.32
Stainless Steel	0.20
Inconel	0.40
Hastelloy	0.37

$$ECSA\ (nominal)\ of\ PTLs = \frac{C_{dl}(obtained\ from\ EIS\ at\ 1.5\ V\ for\ PTLs)}{C'_{dl}(obtained\ from\ EIS\ at\ 1.5\ V\ for\ foils)} \quad (1)$$

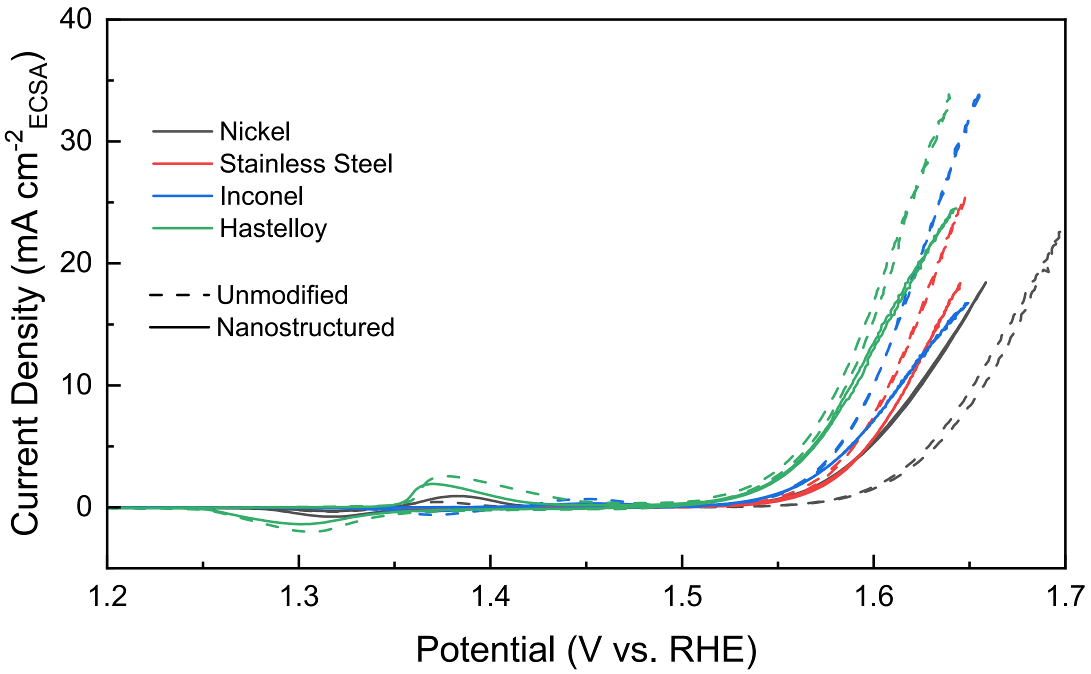

Figure S7: CV curves of PTLs normalized by ECSA. The performance of unmodified PTLs is shown using dashed lines, and the performance of nanostructured PTLs is shown using solid lines

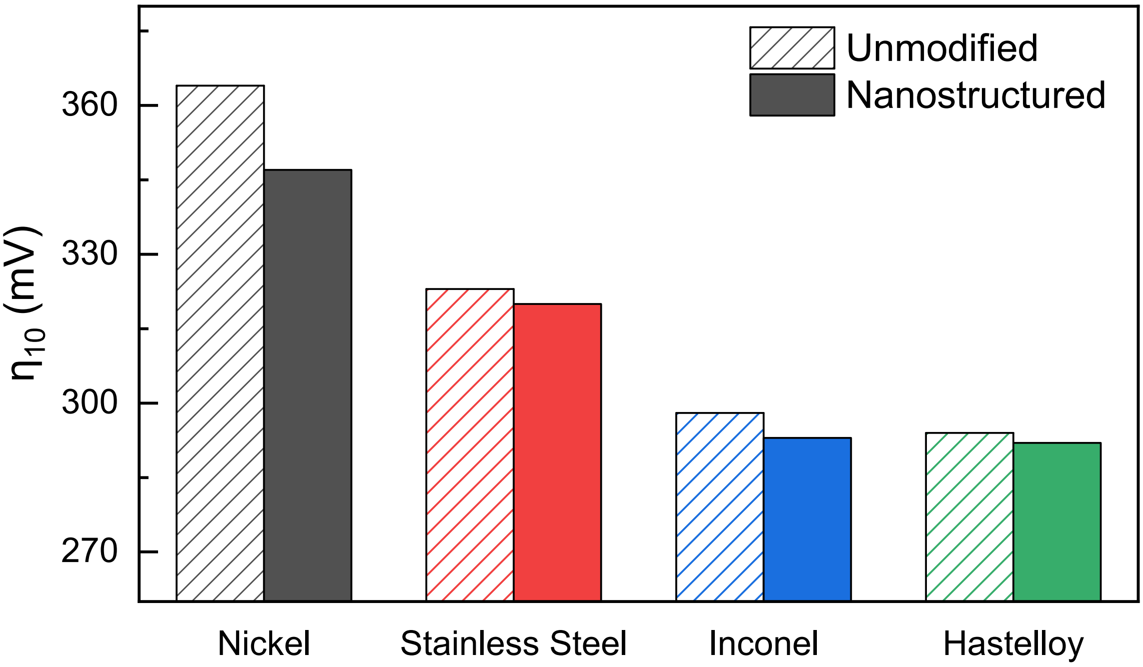

Figure S8: Overpotentials (η) of the unmodified and nanostructured PTLs at 10 mA cm^{-2}

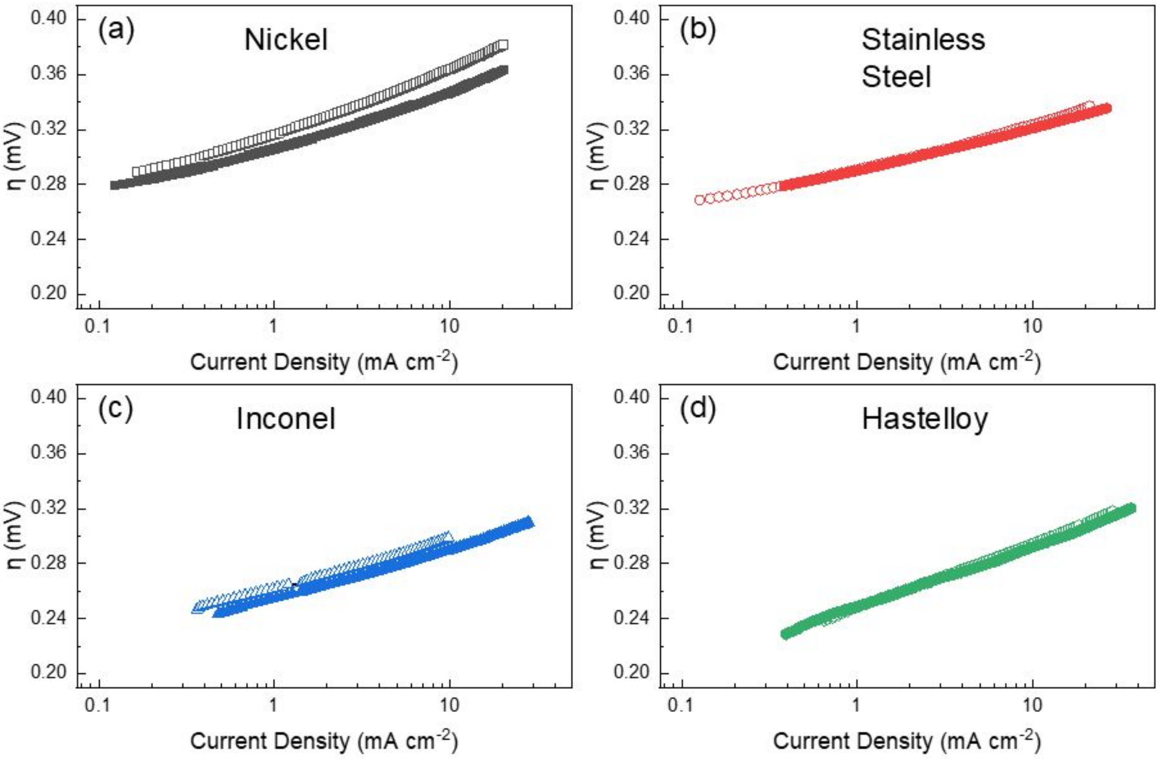

Figure S9: Comparison of the Tafel slopes of the unmodified and nanostructures PTLs, a) Nickel, b) Stainless Steel, c) Inconel, and d) Hastelloy. The open symbols denote the unmodified PTLs and the closed symbols are for the nanostructured PTLs.

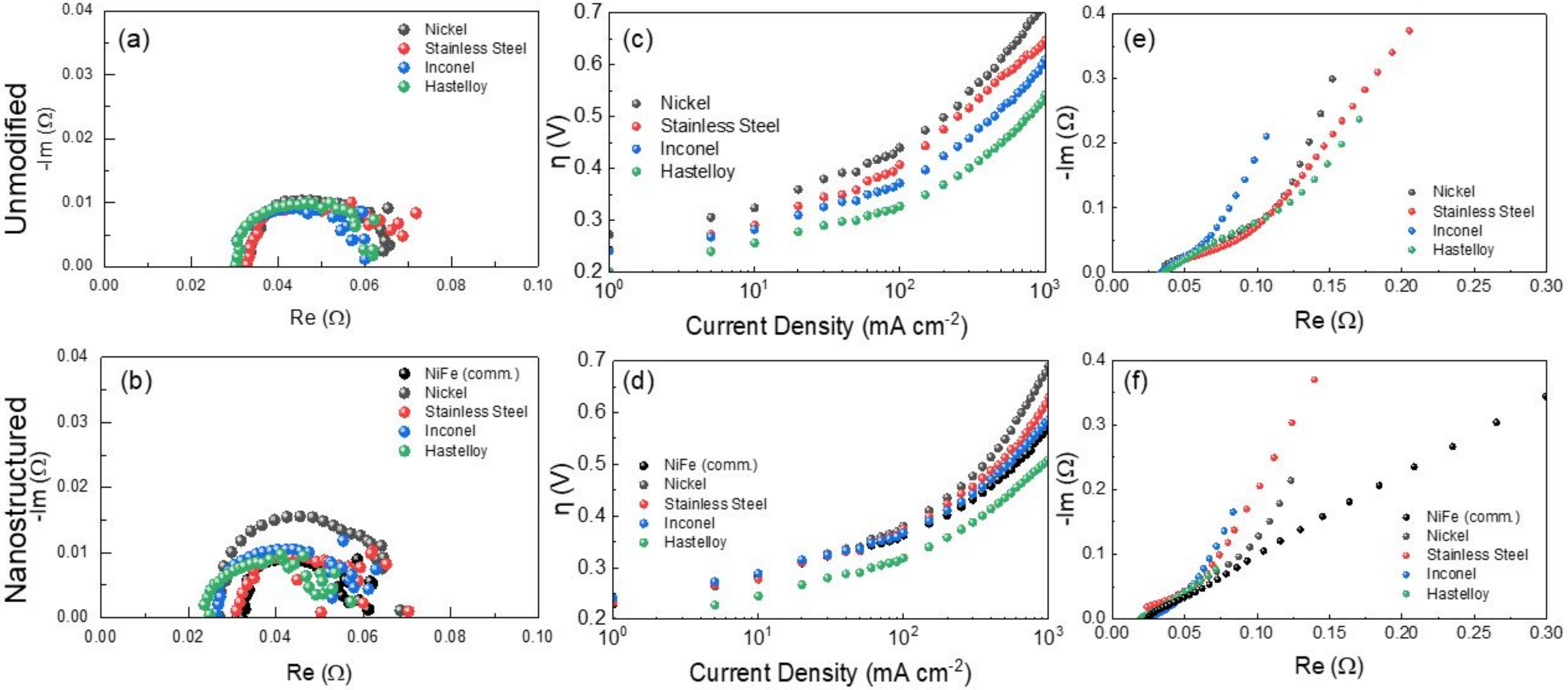

Figure S10: EIS of a) unmodified and b) nanostructured PTLs at 1 A cm^{-2}, HFR-corrected polarization curves of unmodified PTLs (c) and nanostructured PTLs (d), EIS at 1.4 V to determine the catalyst layer resistance of unmodified PTLs (e) and nanostructured PTLs (f)

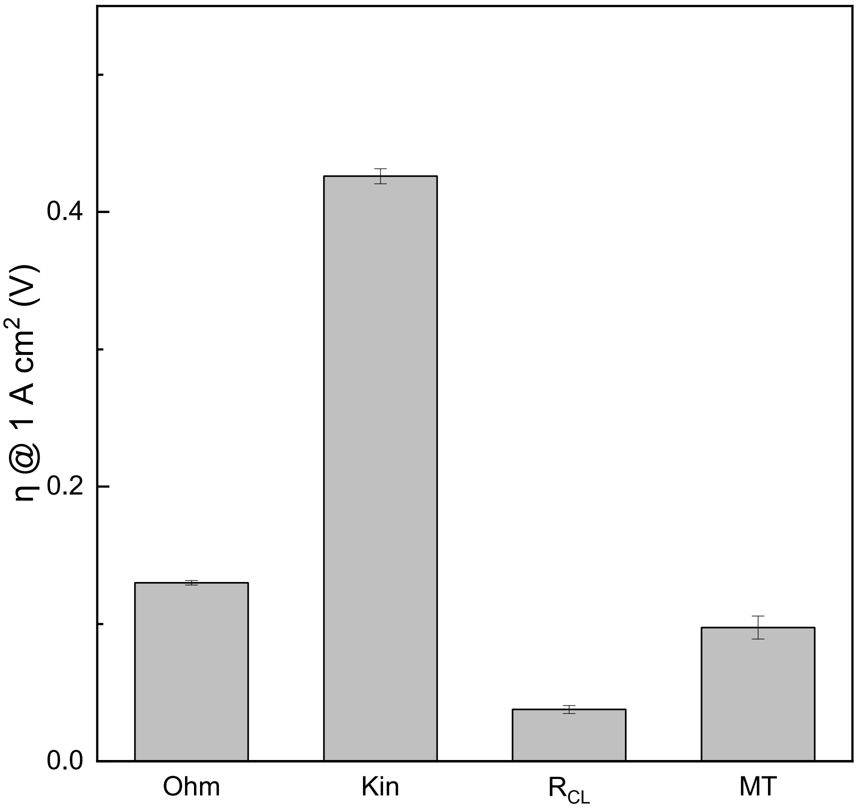

Figure S11: Voltage breakdown analysis at 1 A cm^{-2} of commercial NiFe oxide coated on Stainless Steel PTL

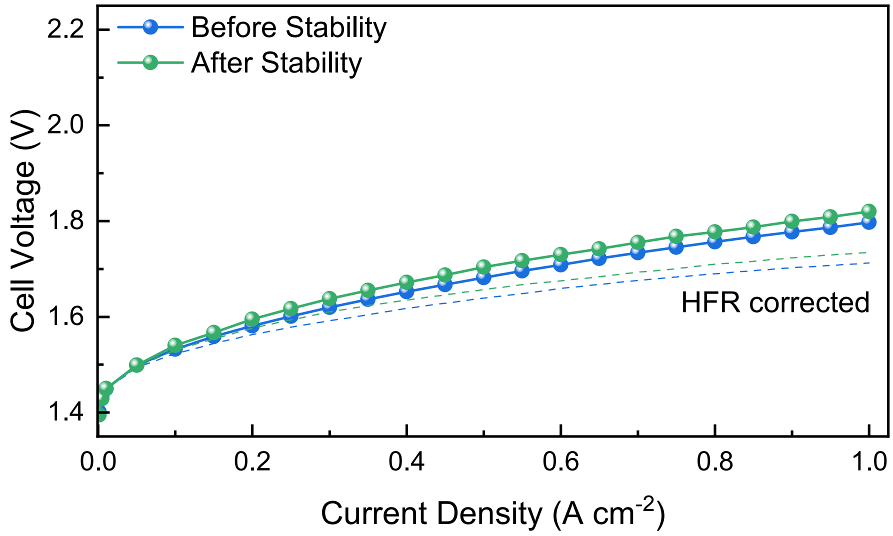

Figure S12: Polarization curve of nanostructured Hastelloy PTL before and after 500 h stability test

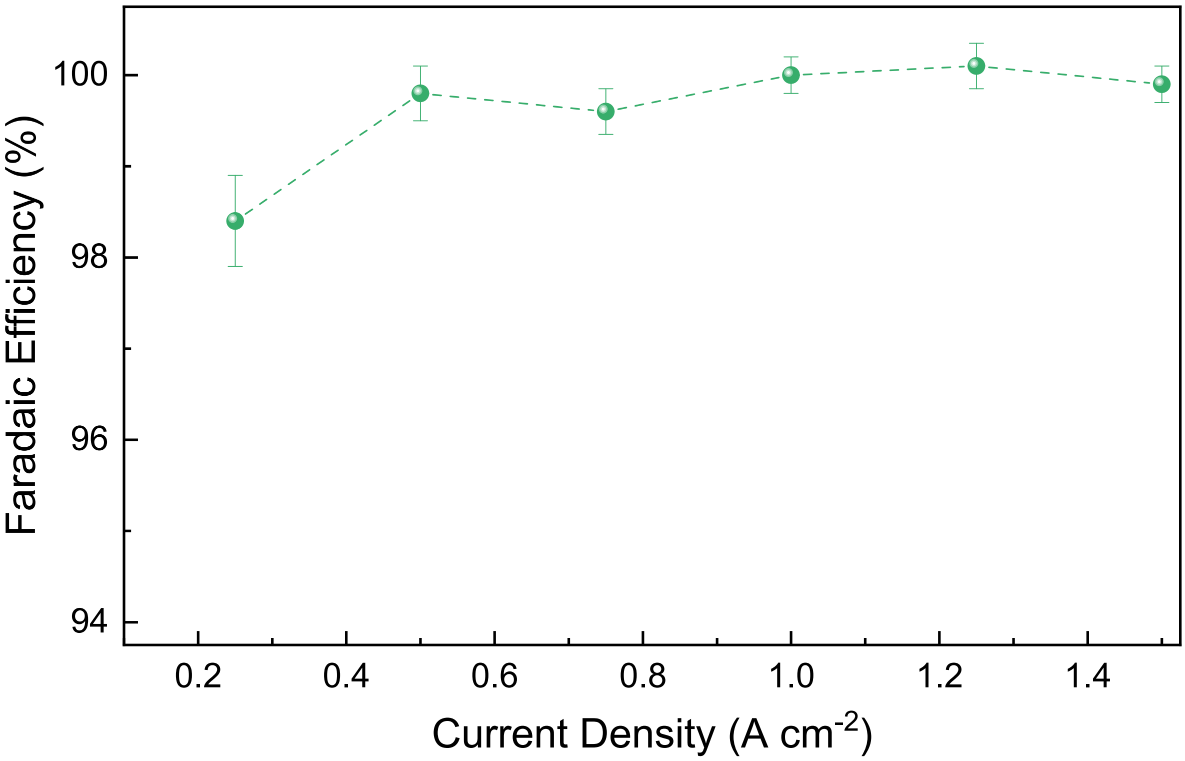

Figure S13: Faradaic efficiency measurement for OER at 50 ˚C using a nanostructured Hastelloy PTL anode

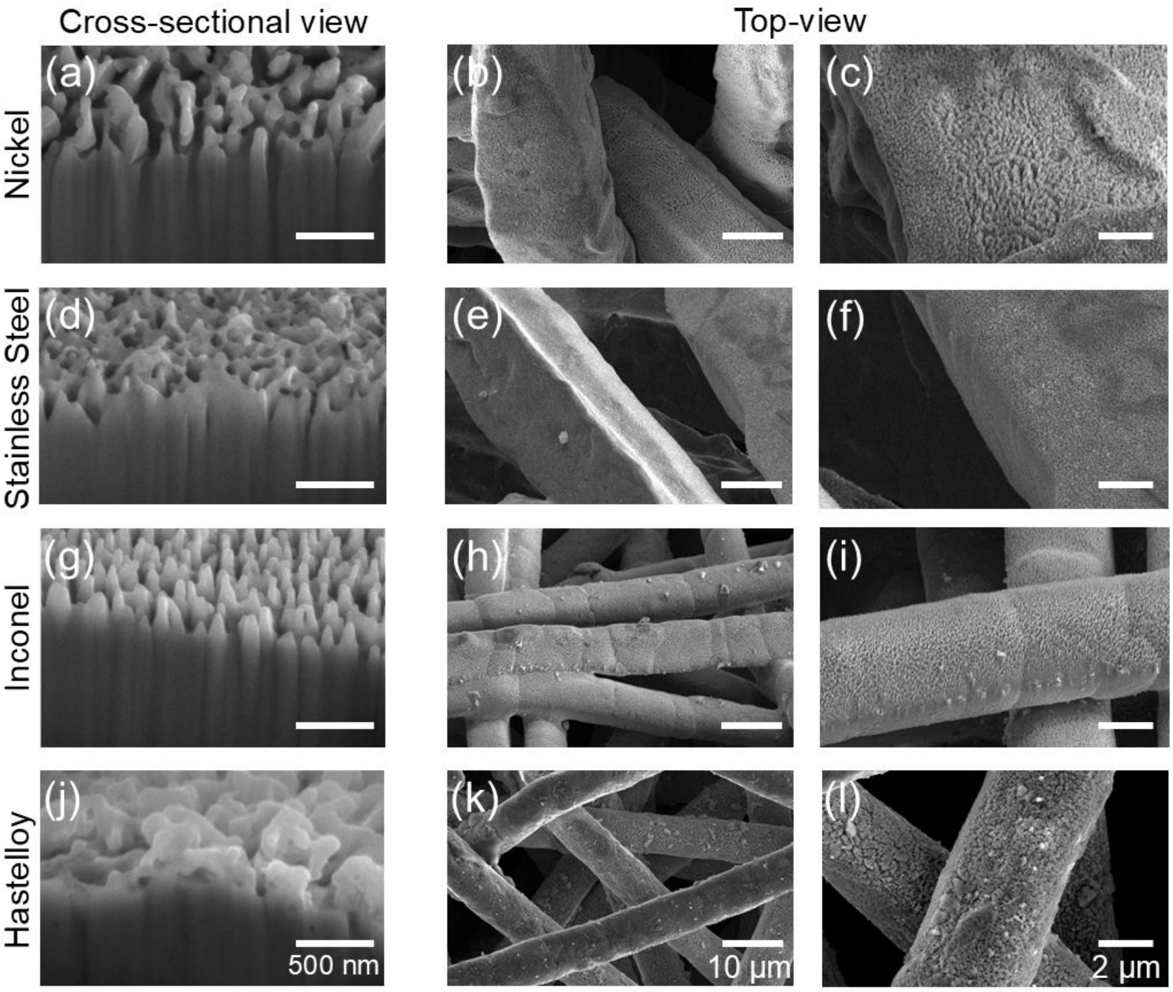

Figure S14: SEM images of nanostructured PTLs after OER. The leftmost column shows the thickness of nanostructures of various PTLs after OER, a) Nickel, d) Stainless Steel, g) Inconel, and j) Hastelloy. The middle column and the right column show the morphology of the nanostructured PTLs after OER at low and high magnifications.

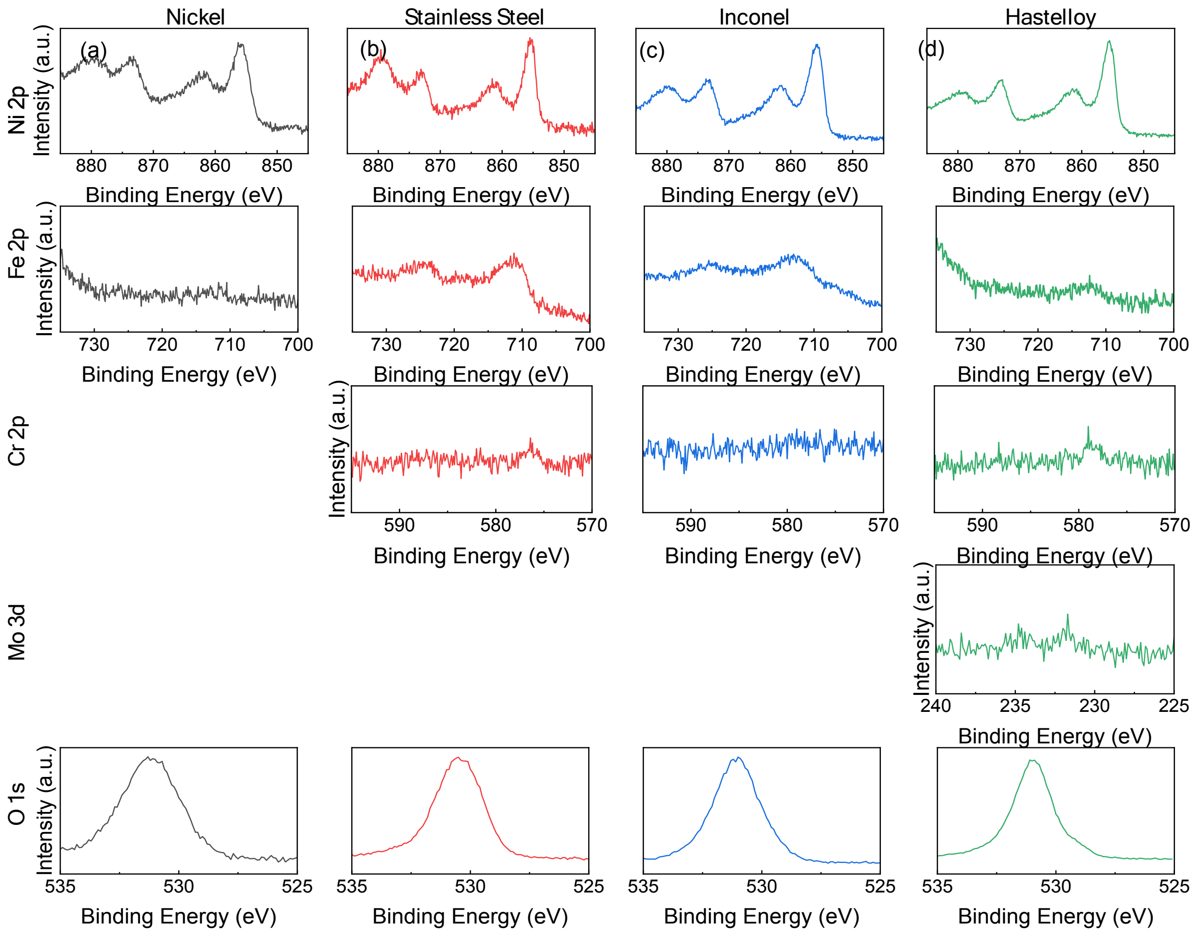

Figure S15: XPS measurements of nanostructured PTLs after OER. The PTL description is given in columns a-d, and the respective spectra are denoted on the left side.

Table S2: Elemental composition of PTLs after OER

Description	Elemental composition (atomic %)				
	Ni	Fe	Cr	Mo	Bal.
Nickel	99.9	0.1			
Stainless Steel	9	67	18	1	5
Inconel	52.5	13.4	25.3	-	8.8
Hastelloy	54.8	3.6	25.7	9.3	6.6

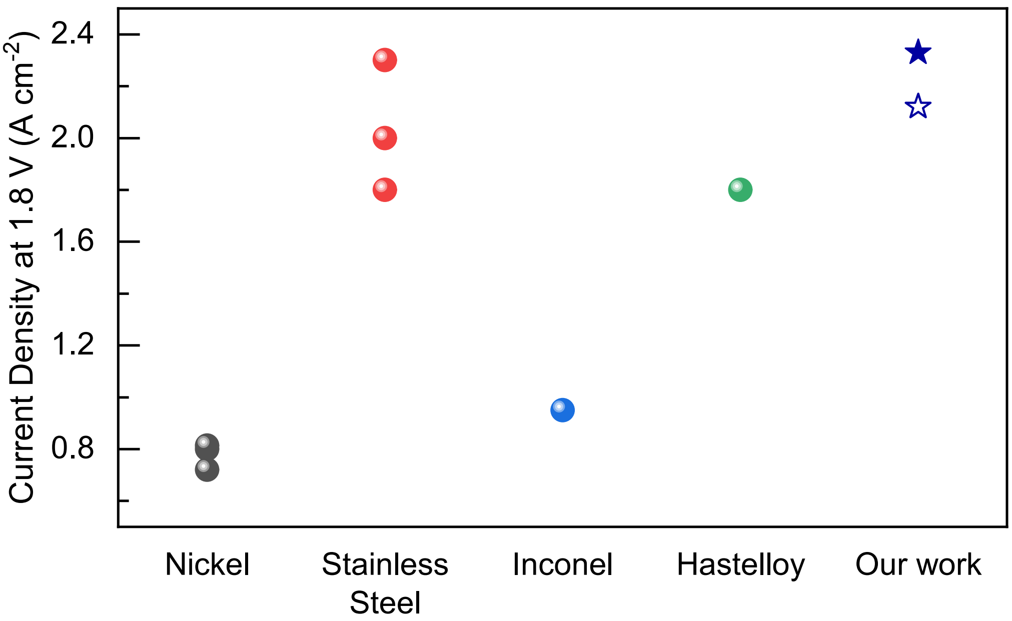

Figure S16: Comparison of the performance of standalone PTLs at 1.8 V using 1 M KOH electrolyte and 80 ˚C cell temperature. Open star symbol represents the performance of unmodified Hastelloy PTL, and closed star symbol represents the nanostructured Hastelloy PTL. The performance of other PTLs is taken from references [3,5,6]

References

(1) *Upgraded Pilot-PSI (UPP) | Dutch Institute for Fundamental Energy Research*. https://www.differ.nl/facilities/upgraded-pilot-psi (accessed 2023-04-10).

(2) Ranade, A.; Lao, M.; Timmer, R. H. M.; Zoethout, E.; van Eck, H. J. N.; Tsampas, M. N. Plasma-Driven Synthesis of Self-Supported Nickel-Iron Nanostructures for Water Electrolysis. *Adv. Mater. Interfaces* **2023**, *10* (34), 2300486. https://doi.org/10.1002/admi.202300486.

(3) Kreider, M. E.; Yu, H.; Osmieri, L.; Parimuha, M. R.; Reeves, K. S.; Marin, D. H.; Hannagan, R. T.; Volk, E. K.; Jaramillo, T. F.; Young, J. L.; Zelenay, P.; Alia, S. M. Understanding the Effects of Anode Catalyst Conductivity and Loading on Catalyst Layer Utilization and Performance for Anion Exchange Membrane Water Electrolysis. *ACS Catal.* **2024**, *14* (14), 10806–10819. https://doi.org/10.1021/acscatal.4c02932.

(4) Padgett, E.; Bender, G.; Haug, A.; Lewinski, K.; Sun, F.; Yu, H.; Cullen, D. A.; Steinbach, A. J.; Alia, S. M. Catalyst Layer Resistance and Utilization in PEM Electrolysis. *J. Electrochem. Soc.* **2023**, *170* (8), 084512. https://doi.org/10.1149/1945-7111/acee25.

(5) Tricker, A. W.; Ertugrul, T. Y.; Lee, J. K.; Shin, J. R.; Choi, W.; Kushner, D. I.; Wang, G.; Lang, J.; Zenyuk, I. V.; Weber, A. Z.; Peng, X. Pathways Toward Efficient and Durable Anion Exchange Membrane Water Electrolyzers Enabled By Electro-Active Porous Transport Layers.

Adv. Energy Mater. **2023**, 2303629. https://doi.org/10.1002/AENM.202303629.

(6) Kreider, M. E.; Maldonado Santos, A. R.; Clauser, A. L.; Sweers, M. E.; Hu, L.; Volk, E. K.; Chan, A.-L.; Sugar, J. D.; Alia, S. M. Porous Transport Layers for Anion Exchange Membrane Water Electrolysis: The Impact of Morphology and Composition. *ACS Electrochem.* **2025**. https://doi.org/10.1021/acselectrochem.4c00207.
